# Isolating the effects of storm events on arctic aquatic bacteria: temperature, nutrients, and community composition as controls on bacterial productivity

**DOI:** 10.3389/fmicb.2015.00250

**Published:** 2015-03-31

**Authors:** Heather E. Adams, Byron C. Crump, George W. Kling

**Affiliations:** ^1^Department of Ecology and Evolutionary Biology, University of MichiganAnn Arbor, MI, USA; ^2^College of Earth, Ocean and Atmospheric Science, Oregon State UniversityCorvallis, OR, USA

**Keywords:** aquatic, arctic, bacterial production, diversity, experiment, nutrients, 16S rRNA, temperature

## Abstract

Storm events can pulse nutrients and carbon from soils and provide an important subsidy to food webs in oligotrophic streams and lakes. Bacterial nutrient limitation and the potential response of stream aquatic bacteria to storm events was investigated in arctic tundra environments by manipulating both water temperature and inorganic nutrient concentrations in short (up to 4 days) and long duration (up to 2 weeks) laboratory mesocosm experiments. Inorganic N and P additions increased bacterial production (^14^C-labeled leucine uptake) up to seven times over controls, and warmer incubation temperatures increased the speed of this response to added nutrients. Bacterial cell numbers also increased in response to temperature and nutrient additions with cell-specific carbon uptake initially increasing and then declining after 2 days. Bacterial community composition (BCC; determined by means of 16S denaturing gradient gel electrophoresis fingerprinting) shifted rapidly in response to changes in incubation temperature and the addition of nutrients, within 2 days in some cases. While the bacteria in these habitats responded to nutrient additions with rapid changes in productivity and community composition, water temperature controlled the speed of the metabolic response and affected the resultant change in bacterial community structure, constraining the potential responses to pulsed nutrient subsidies associated with storm events. In all cases, at higher nutrient levels and temperatures the effect of initial BCC on bacterial activity was muted, suggesting a consistent, robust interaction of temperature, and nutrients controlling activity in these aquatic systems.

## Introduction

Nutrient limitation of bacteria occurs in a wide variety of aquatic habitats including wetlands, rivers, lakes, and marine habitats (Morris and Lewis, 1992; Mohamed et al., 1998; Waiser, 2001; Castillo et al., 2003; Kuosa and Kaartokallio, 2003; Granéli et al., 2004). Bacterial growth in freshwater habitats of arctic Alaska is likely to be nutrient limited because of low nutrient supply, but bacteria in these environments must also contend with low temperatures that may limit bacterial growth (White et al., 1991; Panzenbock et al., 2000) and interact with nutrient limitation. For example, the bacterial response to nutrients has been linked to seasonal variations in temperature, and the degree of nutrient limitation can vary with season and water temperature (Hall et al., 2009; Hoikkala et al., 2009). Additionally, direct testing of temperature and nutrient effects often indicates co-limitation by these factors (Wiebe et al., 1992; Pomeroy and Wiebe, 2001; Vrede, 2005; Mindl et al., 2007; Säwström et al., 2007).

In the Arctic, pulses of nutrients flushed from soils during storm events act as important subsidies to oligotrophic lakes and streams (Stieglitz et al., 2003). Because bacteria are limited in their ability to retain nutrients (Vadstein, 2000), pulsed nutrient supply can suspend bacterial nutrient limitation at least for the duration of the pulse. Rapid changes in temperature, nutrients, and the quality and quantity of organic matter associated with storm pulses may limit the ability of bacterial communities to shift to an optimal activity for a given resource supply when environmental variability is on the same time scale as their growth rate. Therefore, examining the effects of these pulses on bacteria on the time scale of storm events may provide a mechanistic understanding of the interaction of temperature and nutrient limitation in any aquatic habitat that experiences pulsed nutrient supply (e.g., storm events). In addition, separating the individual influences of temperature and nutrients from their interactive effect is required to fully examine the impacts of these drivers on bacterial activity and composition in natural habitats.

Bacterial communities contain populations with different metabolic capabilities and thus different potential responses to changing temperature and nutrients. Shifts in community composition occur as populations change in dominance in response to different optimal conditions or differential mortality. For example, several investigators have found correlations between bacterial community composition (BCC) and resource supply in natural habitats (Pearce, 2005; Yannarell and Triplett, 2005; Xing and Kong, 2007). Previously rare populations can increase in abundance in response to a new substrate (Szabo et al., 2007; Nelson and Carlson, 2011; Crump et al., 2012) and the now altered community may be able to access different substrates and may have different nutrient requirements, affecting both community structure and function. What has not yet been determined is the interaction of temperature and nutrients with bacterial community structure in natural habitats. Individually, warmer temperatures and increased nutrient concentrations can increase bacterial productivity (White et al., 1991; Ram and Sime-Ngando, 2008) and, potentially, select for communities that can reproduce fastest under those conditions. However, in highly variable environments, bacterial communities may be constrained to a short-term physiological response, particularly if temperature and nutrients select for different bacterial populations, resulting in a relatively static community because populations lack the time to respond.

An initial observational study of bacterial production (BP) during storm events indicated covariance of several potential drivers of activity such as temperature and nutrients. Thus, in this study, we conducted experiments with natural bacterial communities to isolate the influence of temperature and nutrient supply on bacterial activity, growth rate, and community structure. We hypothesized that productivity of bacterial communities in ultra-oligotrophic arctic streams and lakes would be elevated mainly by temperature or nutrients based on the natural environmental characteristics (e.g., DOM, temperature, and nutrients) the communities usually experience. We anticipated that communities from sites with high quality algal organic matter and more constant temperature, such as lake outlets, would be less nutrient limited and more strongly affected by temperature. Conversely, communities at sites with low quality terrestrial organic matter and more variable temperatures, such as headwater streams, would respond more strongly to nutrients than temperature. In all cases we found that nutrient treatments approximating maximum natural concentrations had a larger impact on BP than did elevated temperature, although warmer incubation temperatures increased the speed of this response to added nutrients; this suggests a robust interaction of temperature and nutrients controlling bacterial activity in these aquatic systems.

## Materials and Methods

### Study Site

**FIGURE 1:**
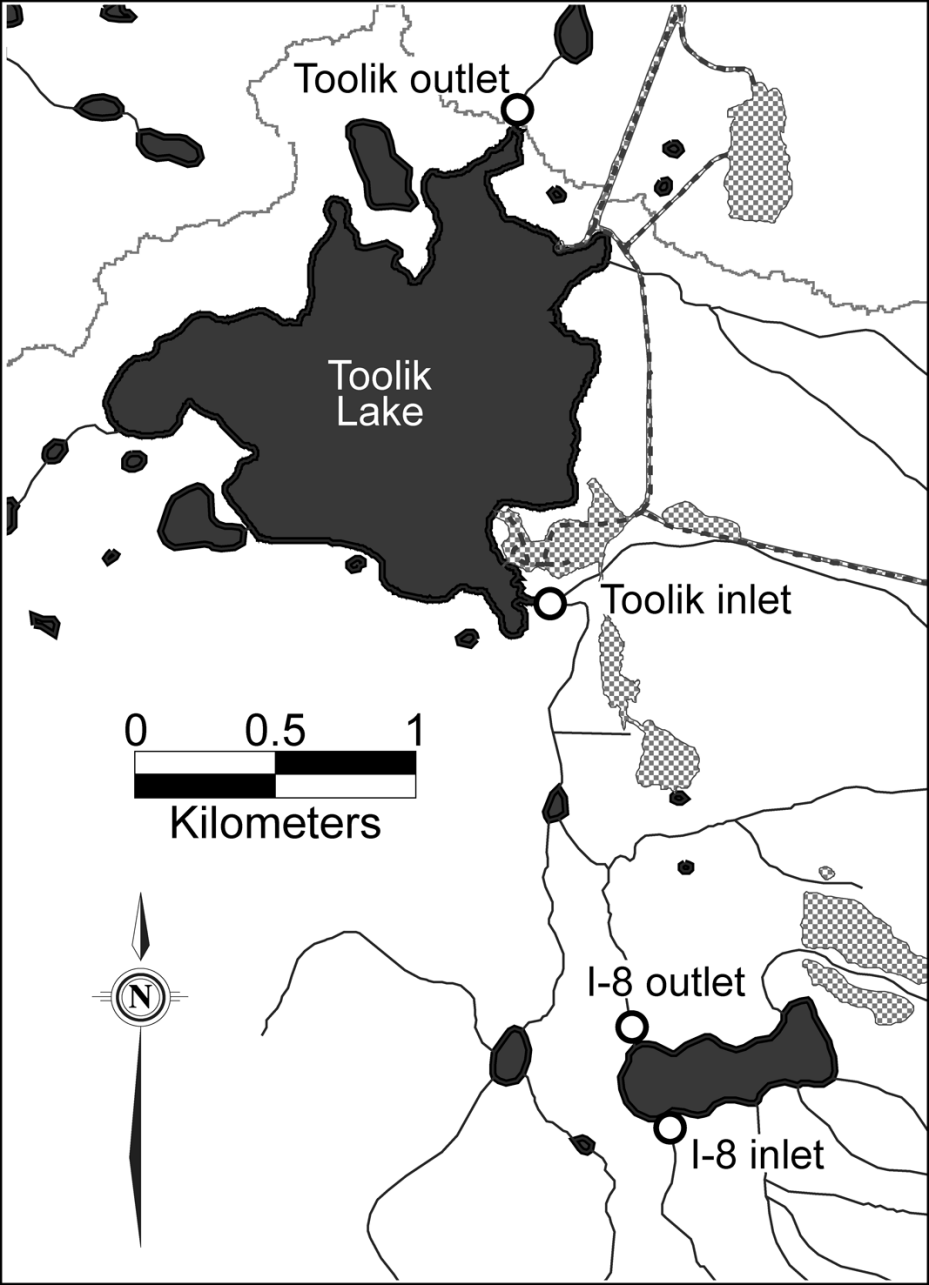
**Sampling locations at Lake I-8 and Toolik Lake, Alaska**. Hatched areas are gravel pads, dashed lines are roads, black lines are streams, and the gray line is the northern edge of the watershed of Toolik Lake.

Sites are located on the north slope of the Brooks Range, Alaska, at the Toolik Field Station (68∘38′N, 149∘36′W). Samples were collected from the inlet and outlet of lakes I-8 and Toolik. Toolik Lake is a multi-basin lake, draining a catchment of 66.9 km^2^, and has a single outlet. Two kilometers upstream of the main Toolik inlet stream is an 18-ha lake, Lake I-8, which has a large headwater stream inlet, I-8 inlet, and a single outlet, I-8 outlet (**Figure 1**).

For summers 2003–2007, average water temperatures were 9.4 and 12.3∘C for Lake I-8 inlet and outlet, respectively, and 11.3 and 13.8∘C for Toolik inlet and outlet, respectively (Adams et al., 2010). All of the lakes in the Toolik Lake catchment are oligotrophic, with mean primary productivity of ∼3.2 μmol C/L/day and mean chlorophyll *a* (chl *a*) concentrations of ∼1.0 μg/L (Kling et al., 2000). I-8 outlet had consistently greater summer concentrations of chl *a* than the I-8 inlet (average of 1.0 μg/L versus 0.31 μg/L, respectively; Supplementary Table S1). Similarly, Toolik Lake, and thus Toolik outlet, had a greater chl *a* concentration than Toolik inlet (average of 1.36 μg/L versus 0.55 μg/L; Kling et al., 2000). All sites had low average concentrations of NH_4_ (<0.8 μM) and PO_4_ (<0.08 μM); however, storm-related pulses of higher concentration and loading did occur (Supplementary Table S1). Both I-8 inlet and Toolik inlet had higher mean concentrations of NO_3_ than I-8 outlet and Toolik outlet (Supplementary Table S1). There are frequently 2–3 storm events during the summer season, which begins after snow-melt runoff in May.

### Field Measurements

In order to detect patterns of bacterial response to natural variations in temperature and nutrient concentrations, temperature, dissolved organic carbon (DOC), inorganic nutrients, and BP were measured weekly in Lake I-8 inlet, I-8 outlet, and Toolik inlet from approximately June 15 to August 20, 2003–2007, and was measured five times in Toolik outlet in summer 2004. Chl *a* was sampled 3–21 times at each site (2003–2007) and DOM measurements of Ultraviolet (UV) absorbance, protein, and phenolics were measured at all sites starting in 2004 (through 2007). Temperature at I-8 inlet (2005–2006) and I-8 outlet (2004–2006) was measured continuously during summer with Onset HOBO temperature loggers (Bourne, MA, USA). Stream discharge and temperature were monitored in Toolik inlet using a Stevens PGIII Pulse Generator (Portland, OR, USA) and a Campbell Scientific Model 247 conductivity and temperature probe (Logan, UT, USA) connected to a Campbell Scientific CR510 datalogger. Temperature at all sites, including Toolik outlet, was also measured during sample collection with a digital thermometer (Fisherbrand Traceable; Thermo Fisher Scientific, Waltham, MA, USA).

Dissolved organic carbon concentration was measured in water collected in the field and immediately filtered through GF/F filters (Whatman, GE Healthcare Life Sciences, Pittsburgh, PA, USA) and acidified to pH ∼3.5 and kept cold and dark until analysis on a Shimadzu TOC-5000 instrument (Columbia, SC, USA) using high-temperature, platinum-catalyzed combustion to CO_2_ and infrared detection. UV absorbance of DOM was measured on unfiltered samples using a quartz cell with a 5 cm path length on a Shimadzu 1601-UV scanning spectrophotometer in the wavelength range of 220 to 400 nm, total proteins were measured using a modified Bradford Reagent method (Bradford, 1976), and total phenolics were measured using the Folin Ciocalteu assay (Waterman and Mole, 1994) and comparing samples to humic acid standards. Chl *a* concentration was determined on GF/F filters and corrected for phaeophytin following Kling et al. (2000).

Inorganic nutrient concentrations were measured in water samples filtered through ashed (450∘C, 4 h) GF/F filters (Whatman) upon collection and stored in the dark at 4∘C (NH_4_ and PO_4_) or frozen (NO_3_) until analysis. Ammonium concentrations were determined within 48 h using a fluorometric OPA method modified from Holmes et al. (1999), and phosphate concentrations were determined within 48 h spectrophotometrically using the molybdenum ascorbic acid assay (Murphy and Riley, 1962). Frozen nitrate samples were analyzed on an Alpkem Flow system 3000 Autoanalyzer (Alpkem, Saskatoon, SK, Canada, now OI Analytical, College Station, TX, USA) using flow injection with a cadmium reduction coil method modified from Armstrong et al. (1967).

Bacterial production was measured using ^14^C labeled-leucine uptake following Kirchman (1994) assuming an isotopic dilution of 1 resulting in a conversion factor of 1.55 kg C (mol leu)^-1^. Each measure was calculated from the incubation with ^14^C leucine of three unfiltered 10 mL subsamples, and one 10 mL control killed with trichloroacetic acid (TCA), for ∼3 h before ending with 5% TCA (final concentration). Samples were filtered onto 0.2 μm nitro-cellulose filters, extracted using ice-cold 5% TCA, placed in scintillation vials, dissolved using ethylene glycol monoethyl ether, flooded with Scintisafe scintillation cocktail and counted on a liquid scintillation counter (Packard Tri-Carb 2100TR; Perkin Elmer, Waltham, MA, USA).

### Mesocosm Experiments

Experiments were conducted to test the response of BP and community composition to enhanced nutrient concentrations typical of storm events under different temperature conditions. All experiments used temperature treatments that matched summer mean (12∘C) and high (17∘C) water temperatures. One experiment tested the response of bacteria to low-level nutrient additions over a 4-day period using nutrient concentrations similar to average natural concentrations measured in Toolik inlet during storm events. A second experiment tested the response of bacteria to higher levels of nutrients for up to 2 weeks using nutrient concentrations similar to the maximum natural concentrations measured in Toolik inlet (Supplementary Tables S2 and S3). This high-level nutrient experiment was repeated six more times. In four of these experiments the source of bacterial communities and incubation water was varied. In two experiments the source of bacterial communities was varied.

Each experiment was a factorial design of manipulated temperature (12 and 17∘C) and nutrients with an inoculum of natural bacterial communities (Supplementary Tables S2 and S3). For all experiments, triplicate incubations of each of the four treatments (12 incubations per experiment) were initiated within 4 h of water collection, and all contained by volume 10% of 1.0 μm filtered water (bacterial inoculum) and 90% of 0.2 μm filtered water collected concurrently. All mesocosms were incubated in the dark (to exclude photosynthesis) in incubators or water baths set to treatment temperature ±1∘C. Starting volumes for each experiment varied from 1 to 3 L (Supplementary Table S3) due to the logistical constraints of transporting large volumes of water to the field station. Experiments were conducted in plastic containers that were acid-washed and rinsed with 0.2 μm filtered sample water. Most experiments were conducted in 4 L LDPE cubitainers (Thermo Fisher Scientific) except Experiments 4a,b, which were conducted in 1 L HDPE bottles (Nalgene, Rochester, NY, USA).

#### Experiment 1

**FIGURE 2:**
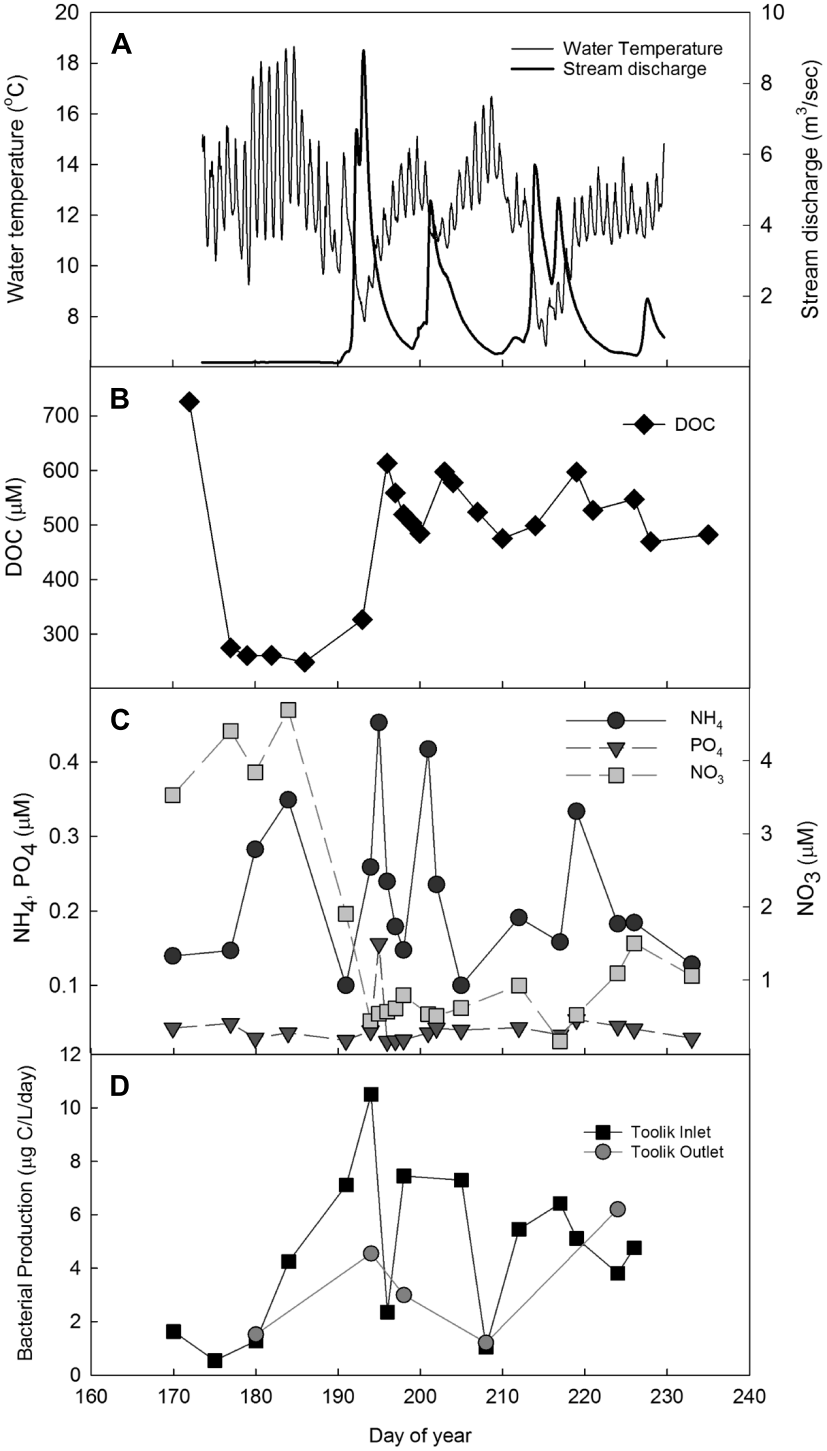
**Toolik inlet stream during summer 2004 from June 8 to August 27. (A)** Temperature (thin line) and stream discharge (thick line); **(B)** DOC (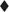); **(C)** NH_4_ (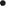), PO_4_ (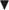), and NO_3_ (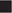); **(D)** BP at Toolik inlet (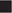) and outlet (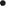).

The experiment conducted with low-level nutrient addition used water and bacteria from Toolik inlet collected on June 22, 2007. This experiment, which was conducted last in our sequence of experiments, was used to determine if there was a threshold of response to added nutrients, and to track the responses of bacterial activity and BCC to temperature and nutrient treatments. Bacterial communities in Toolik inlet represent mixed communities of bacteria from headwater streams and lakes in the Toolik watershed, including nearby Lake I-8 (Crump et al., 2012). Inorganic nutrients were added to nutrient treatments to achieve the average concentration typically observed in Toolik inlet during a storm event (1.5 μM NH_4_NO_3_ and 0.25 μM KH_2_PO_4_; **Figure 2**; Supplementary Table S1). BP was measured in all replicates and treatments at approximately 0, 2, 4, 6, 8, 10, 14, 21, 26, 32, 39, and 49 h, and BP was also measured in the 12∘C treatments at 60, 72, 83, and 98 h. Samples for DNA and cell counts were collected at 26 and 49 h and the 12∘C treatments were also sampled at 72 and 98 h. Sampling of the 17∘C treatments was discontinued after BP stopped increasing.

#### Experiment 2

One experiment with high-level nutrient addition used water and bacteria from Lake I-8 inlet starting on June 27, 2006. This experiment was conducted to track the response of bacterial activity and community composition at different temperatures to the maximum nutrient concentrations typically observed in Toolik inlet during storm events. Inorganic nutrients were added to nutrient treatments to achieve these concentrations (6.4 μM NH_4_NO_3_ and 0.45 μM KH_2_PO_4_). BP was measured at 2, 4, 6, 9, 11, and 14 days, and samples for DNA and cell abundance were collected at 2, 4, 9, and 14 days.

#### Experiments 3a–d

Four high-level nutrient experiments were conducted varying the source of bacterial communities and incubation water starting on July 12, 2005 to examine community-specific responses (that is, the effect of different initial community compositions). These experiments were performed with bacteria and water from Lake I-8 inlet and Lake I-8 outlet in factorial combination. Bacterial communities from Lake I-8 inlet are headwater stream communities with no contribution of bacteria from lakes, whereas communities from Lake I-8 outlet are lake communities (Crump et al., 2007). BP was measured at 2, 4, 6, 9, 12, and 14 days, and samples for DNA and cell abundance were collected at 14 days.

#### Experiments 4a,b

Two high-level nutrient experiments were conducted varying the source of bacterial communities starting on July 18, 2006 to confirm the changes in BCC identified in Experiments 3a–d. One experiment was performed with bacteria and water from Lake I-8 inlet, and the other with bacteria and water from Lake I-8 outlet. Samples for DNA and cell counts were collected at 6 and 11 days. BP was not measured.

### Bacterial Abundance and Community Composition Analyses

Samples for cell counts were preserved with 2.5% of glutaraldehyde (final concentration) and stored at 4∘C until analysis. Samples from 2005 were counted on a FACSCalibur (BD Biosciences, San Jose, CA, USA) flow cytometer following del Giorgio et al. (1996). Sub-samples were stained with SYBR green (Life Technologies, Grand Island, NY, USA) in the dark for a minimum of 15 min (Marie et al., 1997; Lebaron et al., 1998). The concentration of beads in the standard 1 μm bead solution and concentration of cells in multiple confirmatory samples were measured by epifluorescence microscopy. Samples from 2006 and 2007 were counted on a LSR II flow cytometer (BD Biosciences) as described by Ewart et al. (2008) with data acquired in log mode for at least 60 s and until 20,000 events were recorded, with the minimum green fluorescence (channel 200) set as the threshold.

DNA samples were collected from laboratory mesocosms by filtering ∼500 mL of sample through a Sterivex-GP 0.2 μm filter (EMD Millipore, Billerica, MA, USA). Filters were preserved using a DNA extraction buffer as described by Crump et al. (2003) and stored at -80∘C until extraction. DNA was extracted using phenol–chloroform (Crump et al., 2003, 2007) and PCR amplified using 357f with a G-C clamp and 519r universal 16S rDNA bacterial primers on a Bio-Rad thermocycler (Hercules, CA, USA) following (Crump et al., 2003, 2007). DNA was then separated using denaturing gradient gel electrophoresis (DGGE) with an 8% acrylamide gel cast with either a 40 to 60% or 35 to 55% gradient of urea and formamide (Crump et al., 2003, 2007). Gels were run on a CBS scientific DGGE system (Del Mar, CA, USA) for 18 to 24 h at 75 volts and 65∘C. A DGGE ladder, previously constructed from PCR-amplified clones of 16S rRNA genes from Toolik Lake (Crump et al., 2003) was run every six lanes in order to accurately assess the vertical position of bands across each gel.

Imaging of DGGE banding patterns was performed with Quantity One software (Bio-Rad) on a Chemi-Doc gel documentation system (Bio-Rad), gel bands were identified using GelCompar II software (Bionumerics, Applied Maths, Austin, TX, USA) to create a presence–absence matrix as described by Crump and Hobbie (2005). DGGE is capable of detecting bacterioplankton populations that make up at least 0.1 to 0.4% of bacterioplankton in a sample, depending on copy number of rRNA operons per cell and PCR primer specificity (Muyzer et al., 1993; Kan et al., 2006). Each band represents an operational taxonomic unit (OTU) of bacteria, although occasionally multiple sequences may be present within a band (Crump et al., 2003, 2004) or bacteria may differ in a more variable region of the 16S gene; therefore, changes detected here are considered to be a conservative index of shifts of community composition.

### Statistical Analyses

Pairwise similarity values of the DGGE bands were calculated using the Dice equation in order to condense presence–absence data into percent community similarities between samples. PROXCAL was used to create non-metric multi-dimensional scaling (NMDS) graphs of sample similarities. Two-way, between-subjects ANOVA were performed in which percent similarity between samples was designated as the dependent variable with categorical dummy variables indicating the same or different treatment types of incubation temperature or nutrient addition as predictors. Both normal distribution of data and homogeneity of variance were verified using a Shapiro–Wilk test with data log-transformed where necessary. On the two datasets that did not meet ANOVA assumptions following data transformations (temperature and nutrients on day 1), the non-parametric Kruskal–Wallace test was performed to verify the significance of ANOVA results (**Table 1**). All statistical analyses were performed with SPSS (version 17, IBM, Armonk, NY, USA).

**Table 1:** **Experiment 1**.

Dependent variable: community similarity					

		**Significance (*p*-value)**			
	**df**	**1 day**	**2 days**	**3 days**	**4 days**
Corrected model	3	0.051	0.000	0.001	0.020
Intercept	1	0.000	0.000	0.000	0.000
Temperature	1	0.437^∗^	0.002		
Nutrients	1	0.747^∗^	0.001	0.001	0.020
Temperature ^∗^ nutrients	1	0.006	0.163		

		**Degrees of freedom**			
Error		62	62	13	13
Total		66	66	15	15
Corrected total		65	65	14	14

*R Squared = 0.12 (Adjusted R Squared = 0.08) 1 day*

*R Squared = 0.25 (Adjusted R Squared = 0.21) 2 days*

*R Squared = 0.59 (Adjusted R Squared = 0.56) 3 days*

*R Squared = 0.35 (Adjusted R Squared = 0.30) 4 days*

*Results of the tests of significance (ANOVA) for the impact of incubation temperature and low-level nutrient addition on the % similarity of bacterial community composition (BCC) between samples in Experiment 1 at Toolik inlet. The *p*-value for the main effects (temperature and nutrients) and the interaction term is given for each day of the experiment; days 3 and 4 are for low-temperature treatments only. A Kruskal–Wallace test for day 1 data (^∗^ in Table) confirmed non-significance with *p* = 0.907 for temperature and *p* = 0.969 for nutrients. The overall model R-squared is given for each day of the experiment.*

## Results

### Field Measurements: Storm Events, Nutrients, and Bacterial Production

During the summer of 2004, there were three large storm events characterized by rain and subsequent increases in stream discharge (>4 m^3^/s at Toolik inlet; **Figure 2A**). The events occurred on 9–15 July, 18–24 July, and 30 July – 7 August (two combined events). Ammonium concentrations at Toolik inlet spiked either at the initiation of or immediately after each of the three storm events, while phosphate concentrations increased only immediately after the first event; nitrate concentrations were diluted during these events (**Figure 2C**). At Toolik inlet, peaks in BP corresponded with the occurrence of the three storm events (**Figure 2D**). There was also a small increase of BP at the outlet of the lake after the first storm event.

### Mesocosm Experiments – Bacterial Activity

**FIGURE 3:**
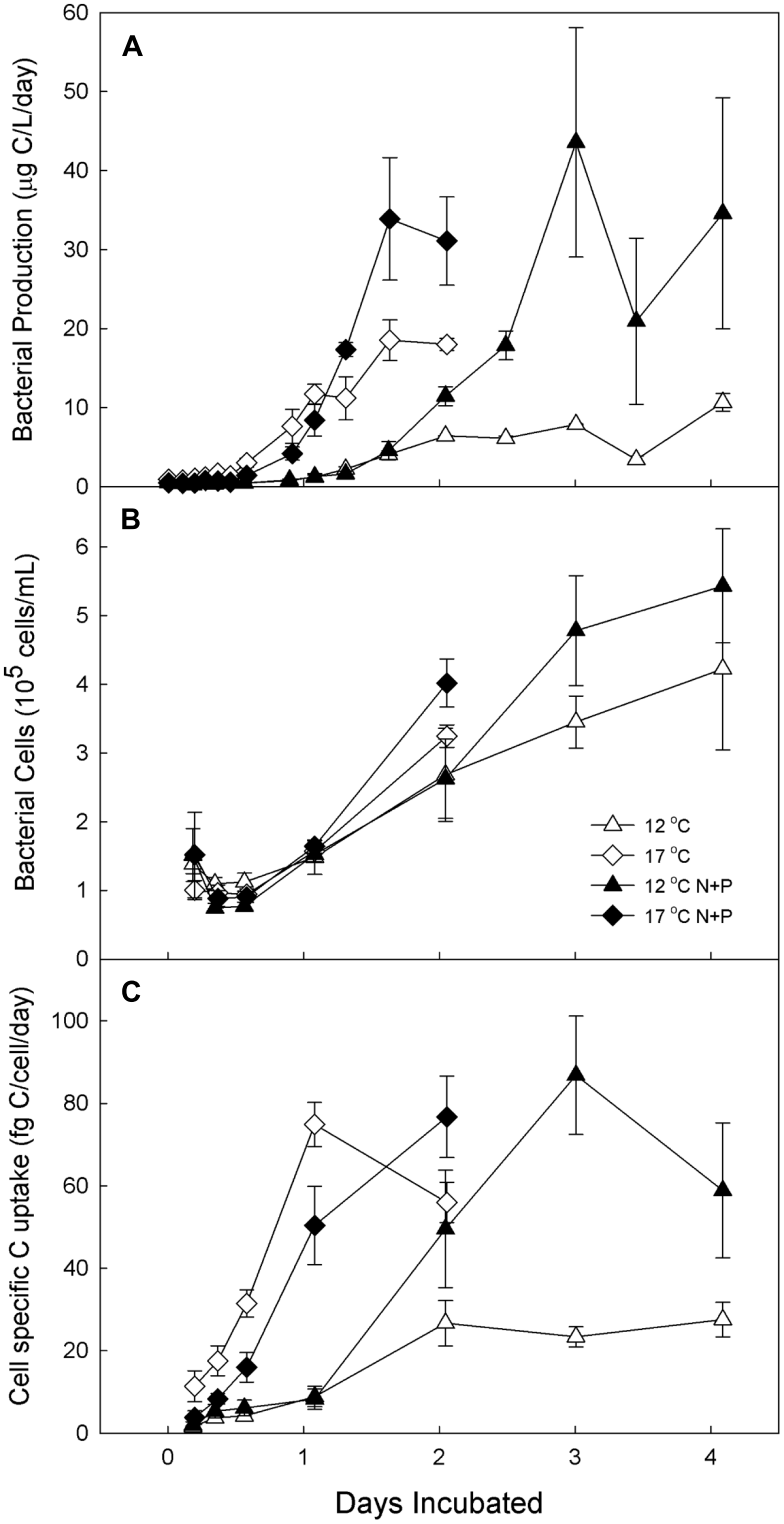
**Experiment 1**. Bacterial production (**A**), cell abundance (**B**), and cell-specific carbon uptake (**C**) in water from Toolik inlet incubated for 4 days at 12 and 17°C with and without low-level nutrient amendments. Error bars are SE of the mean of experimental replicates (*n* = 3). Samples are designated by incubation temperature (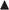 = 12°C and 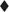 = 17°C) with open symbols for no nutrients added and closed symbols for nutrients added.

BP was elevated by increased temperature and nutrients in all mesocosm experiments, but the timing and magnitude of these treatment responses varied with the concentration of nutrients and, when tested, the initial composition of the bacterial community. When a relatively low level of nutrients was added to water from Toolik inlet (Experiment 1), the bacteria grew more rapidly in the high temperature (17∘C) incubations than the low temperature (12∘C) incubations regardless of nutrient treatment. For example, the 17∘C treatments had ∼10-fold higher BP (**Figure 3A**) and cell-specific carbon uptake (**Figure 3C**) after 1 day in both nutrient treatment and control incubations. At 17∘C, nutrient treatment increased BP by 1.8 times over the control by day 2, but at 12∘C this treatment effect was much greater, increasing BP by 6.1 times over the control by day 3. Similarly, at 12∘C, the cell-specific carbon uptake continued to increase with the nutrient addition when allowed to respond longer than 2 days compared to the 17∘C incubation response at days 1 and 2. Peak BP was similar at both temperatures in the fertilized treatments, but was elevated at 17∘C in the unfertilized treatment. The number of bacterial cells increased roughly exponentially in all treatments after an initial decrease (**Figure 3B**).

**FIGURE 4:**
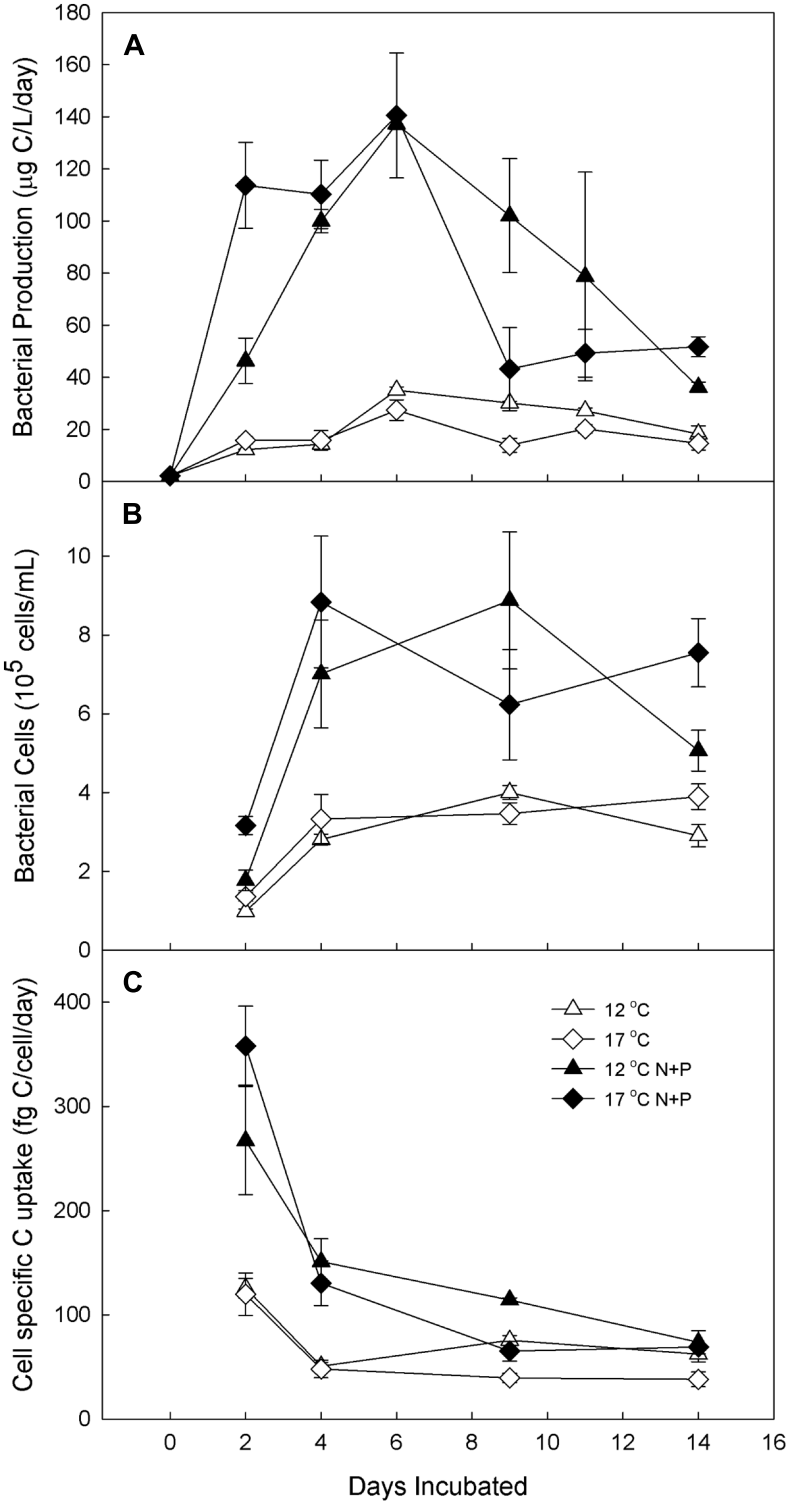
**Experiment 2**. Bacterial production in water from Lake I-8 inlet (**A**) and Lake I-8 outlet (**B**) and cell-specific carbon uptake (**C**) in water from Lake I-8 inlet and incubated for 2 weeks at 12 and 17.C with and without low-level nutrient amendments. Error bars are SE of the mean of experimenta replicates (*n* = 3). Samples are designated by incubation temperature (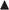 = 12°C and 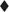 = 17°C) with open symbols for no nutrients added and closed symbols for nutrients added.

When a higher level of nutrients was added to water from Lake I-8 inlet (Experiment 2), the treatments with added nutrients grew more rapidly than unfertilized controls regardless of temperature (**Figure 4**), showing elevated BP and cell-specific carbon uptake after 2 days at both temperatures (**Figures 4A,C**). At both temperatures, high-level nutrient treatment increased BP by 7 times over controls, although this treatment effect was observed more rapidly at 17∘C (2 days) than at 12∘C (4 days). Cell-specific carbon uptake peaked after 2 days in all treatments, similar to Experiment 1 with low-level nutrient treatment, and then decreased during the extended incubation period due to increased cell abundances and decreased BP. Peak BP was the same for both temperatures in the fertilized treatment, but was elevated at 17∘C in the unfertilized controls. Cell abundance, BP, and cell-specific carbon uptake in the fertilized treatments were greater than unfertilized controls and were greater in this experiment compared to those in the low-level nutrient treatments described above. In both experiments (high-level and low-level nutrients), elevated temperatures increased the speed of the BP response to nutrient additions (**Figures 3** and 4) and increased the maximum rate of BP in unfertilized controls, but did not greatly change the maximum rate of BP in fertilized treatments.

**FIGURE 5:**
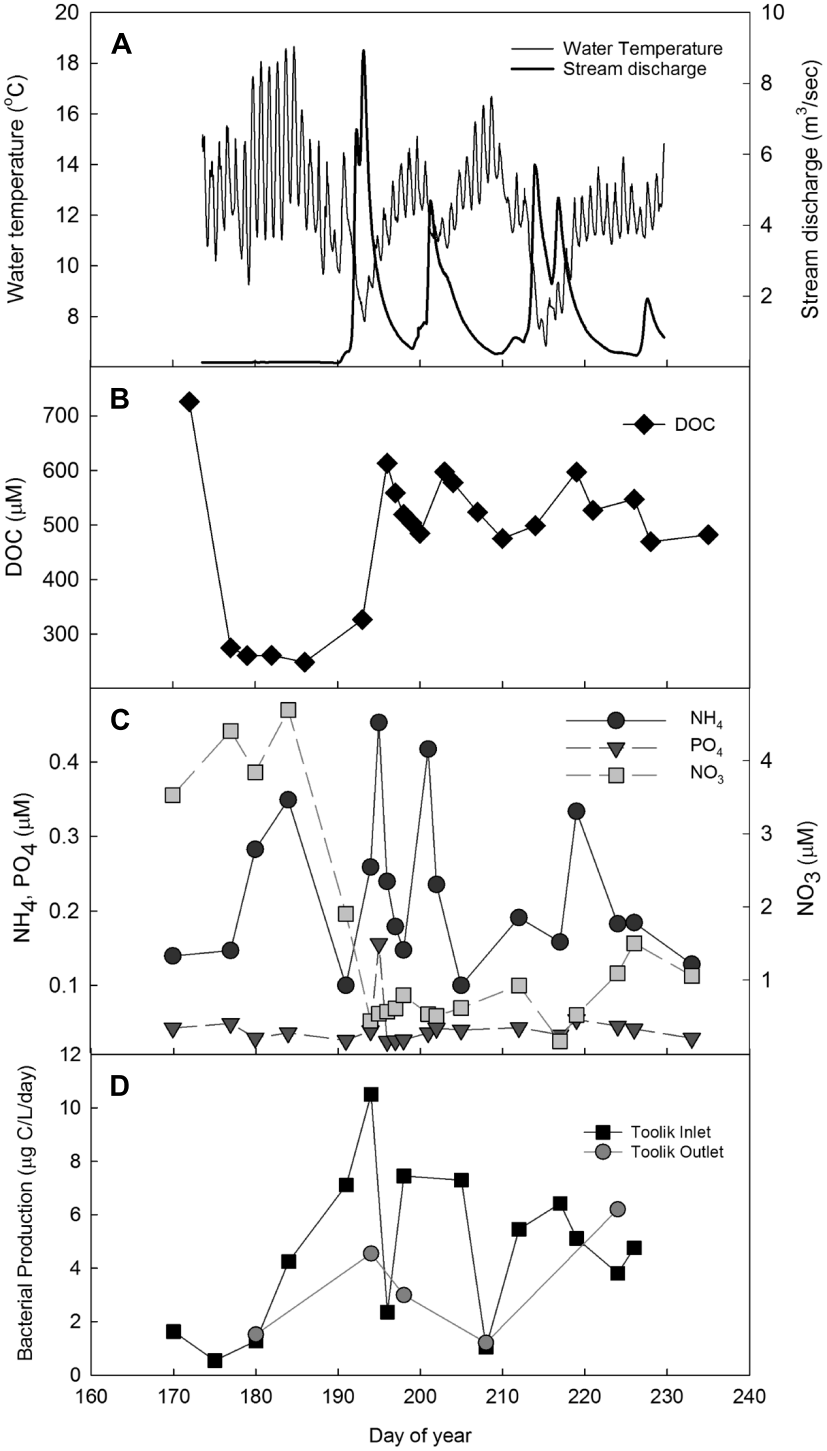
**FIGURE 5. Experiment 3**. Bacterial production in water from Lake I-8 inlet (**A,C**) and Lake I-8 outlet (**B,D**) inoculated with bacterial communities from Lake I-8 inlet (**A,B**) and Lake I-8 outlet (**C,D**) and incubated for 2 weeks at 12° and 17°C with and without high-level nutrient amendments. Error bars are SE of the mean calculated from analytical replicates (*n* = 3). Samples are designated by incubation temperature (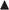 = 12°C and 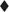 = 17°C) with open symbols for no nutrients added and closed symbols for nutrients added.

This high-level nutrient experiment was repeated with two sources of water and two sources of bacterial communities (Experiments 3a–d) to test the influence of initial BCC and initial water chemistry on treatment responses to temperature and fertilization. Water chemistry was different at the two sites used for these experiments, Lake I-8 inlet and Lake I-8 outlet. Water at Lake I-8 inlet contained 706 μM DOC, 0.76 mg protein L^-1^, 1.16 μM total phenolics, and 0.09 μg chl *a* L^-1^, and had a UV absorbance of 154.3 (scanning from 220 to 400 nm, 5 cm quartz cell). Water at Lake I-8 outlet had lower concentrations of dissolved organics and a much higher concentration of chl *a* (514 μM DOC, 0.57 mg protein L^-1^, 0.65 μM total phenolics, 0.77 μg chl *a* L^-1^, UV absorbance of 98.9). BCC at the two sites also differed, with 50% community similarity at the time of initial collection. The treatment response of BP to fertilization was similar regardless of the source of incubation water or the source of bacterial inocula, increasing more rapidly in the 17∘C treatment but reaching approximately the same maximum productivity at both temperatures in all experiments after 4 days (**Figure 5**). However, in the unfertilized experiments, the response of BP to source waters and temperature was different for the two bacterial communities. The bacterial community from the inlet had elevated BP after 2 days, but the bacterial community from the outlet had lower BP after 2 days; this rate remained low, particularly when the outlet community was incubated in “unfamiliar” water from the inlet stream.

### Mesocosm Experiments – Communities

**FIGURE 6:**
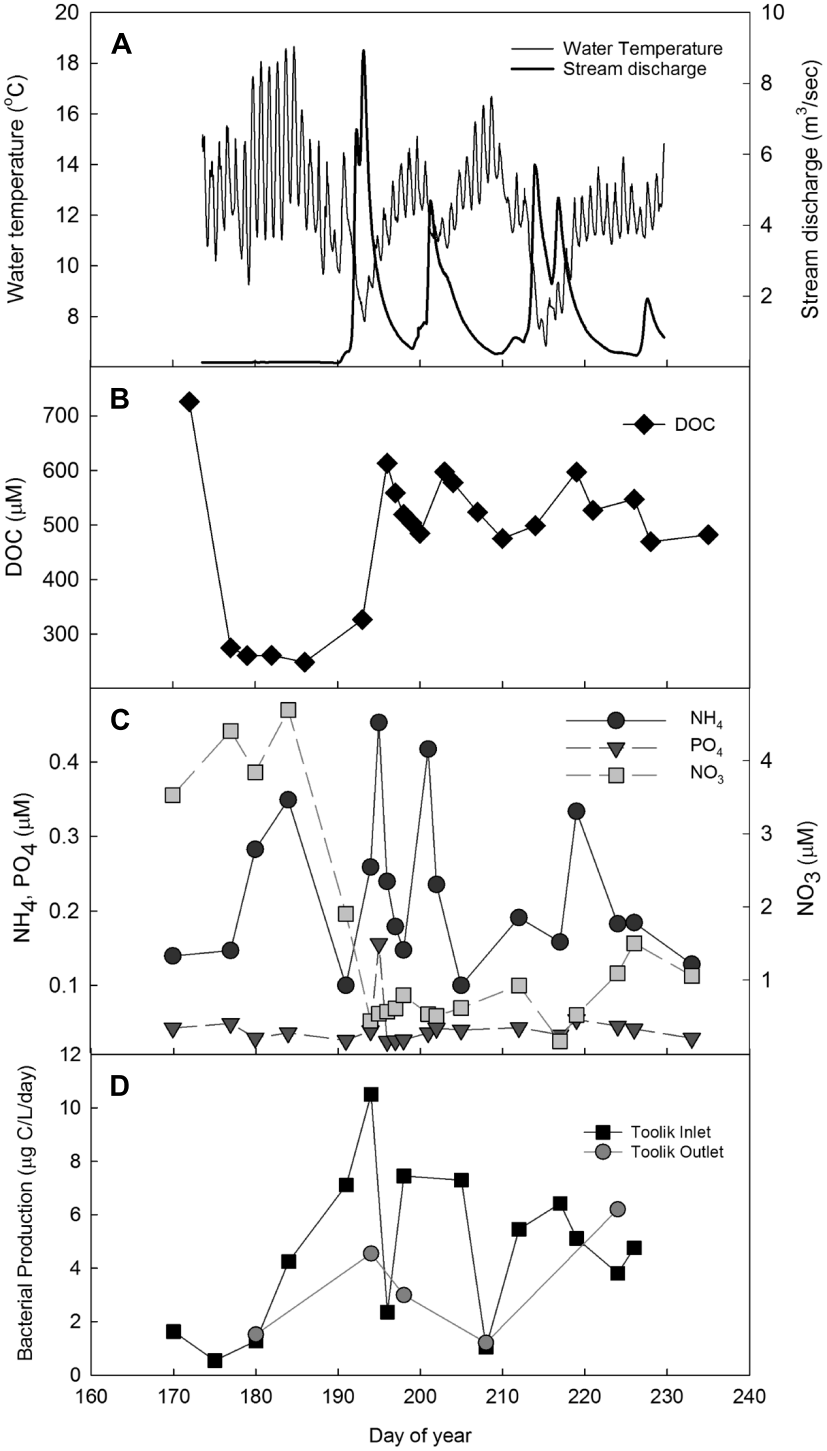
**FIGURE 6. Experiment 1**. Non-metric multi-dimensional scaling (NMDS) plot of community similarity on collection (starting community, day 0) and day 2 of the low-level nutrient experiment. The bacterial community collected from collection site is designated by □. Samples are designated by incubation temperature (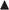 = 12°C and 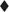 = 17°C) with open symbols for no nutrients added and closed symbols for nutrients added.

Bacterial community composition shifted quickly during regrowth following initial dilution in the experimental mesocosms, and the composition of these communities varied with treatment. After 1 day bacterial communities in the experiment with low-level nutrient additions (Experiment 1) were not significantly different among treatments, but by day 2 a two-way ANOVA of percent similarity between bacterial communities in different treatments indicated that both temperature and nutrient addition were statistically significant indicators of percent similarity (**Table 1**). The pairwise similarities between fertilized and unfertilized treatments at 12∘C declined steadily from 85–76 to 68–64% over the 4 days (**Figure 6**; Supplementary Figure S1), compared to the relatively constant and high pairwise similarities within replicates of the same treatment over the same time period (95, 82, 92, 93%, days 1–4).

**FIGURE 7:**
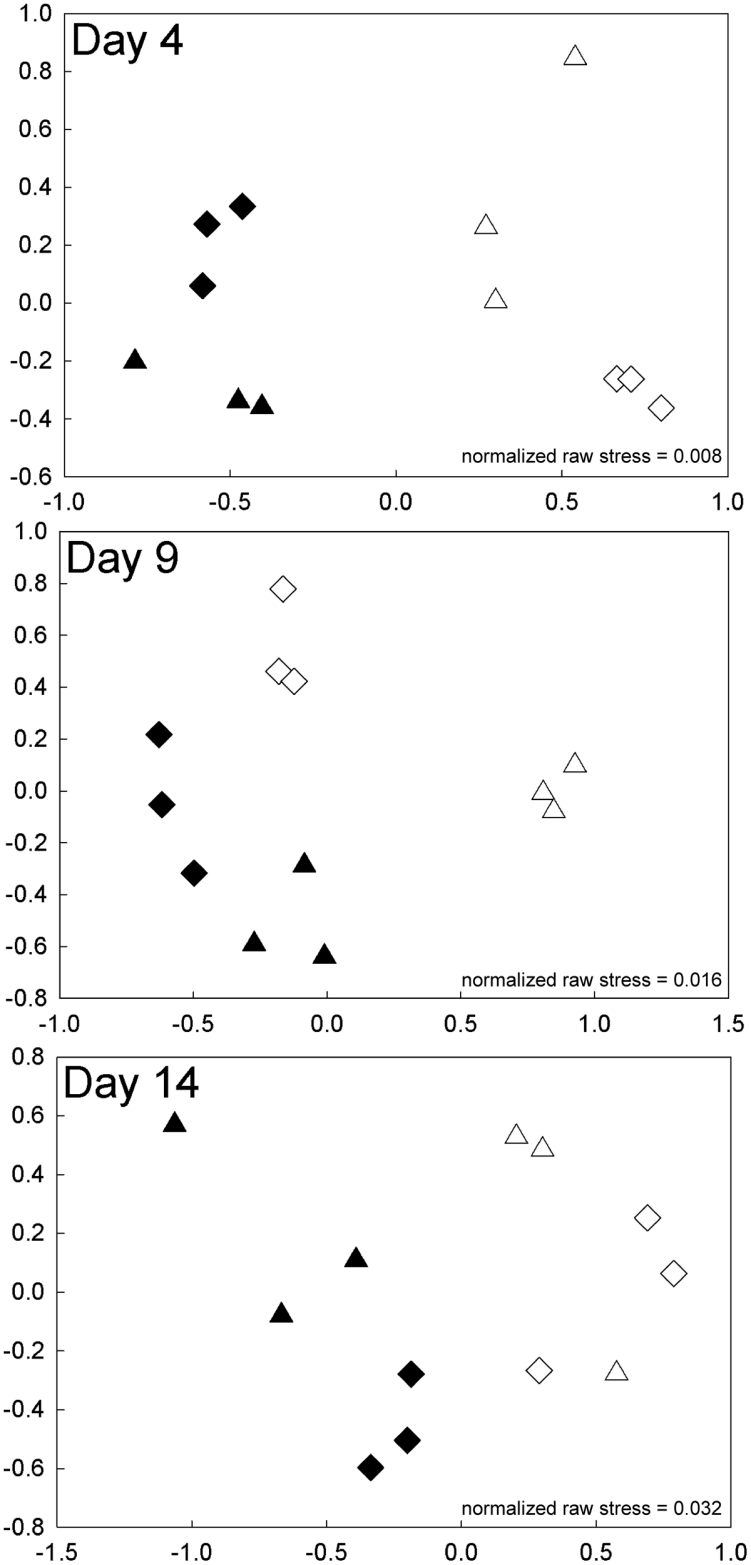
**FIGURE 7. Experiment 2**. NMDS plots of bacterial community composition on days 4, 9, and 14 of the high-level nutrient experiment. Samples are designated by incubation temperature (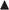 = 12°C and 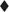 = 17°C) with open symbols for no nutrients added and closed symbols for nutrients added.

Similar patterns in BCC were found in the experiment with high-level nutrient additions to water from Lake I-8 inlet (Experiment 2). BCC clustered by nutrients and temperature in the NMDS analysis for days 2, 4, 9, and 14 (**Figure 7**; Supplementary Figure S1). Nutrients and temperature were statistically significant predictors of community similarity for all time points during the incubation, but the interaction term between temperature and nutrients ceased to be significant on day 14 (**Table 2**). As in the experiment with low level nutrient additions, the average pairwise similarities between fertilized and unfertilized treatments at 12∘C declined over time from 79–71 to 54–59% at 2, 4, 9, and 14 days, respectively, while pairwise similarities within replicates of each treatment remained similar (92, 82, 88, 81% over the same time period). At 17∘C there was also a substantial change in BCC when nutrients were added (mean difference between controls and nutrient additions of 18 ± 3.6%, SE), but unlike at 12∘C there was little change over time. The shift in BCC at 17∘C occurred very rapidly, and within 2 days the pairwise similarity between fertilized and unfertilized treatments was only 69%, and this value remained steady over the 14 days experiment (69, 67, 64, 67% at 2, 4, 9, and 14 days, respectively; **Figure 7**; Supplementary Figure S1).

**Table 2:** **Experiment 2**.

Dependent variable: community similarity					

		**Significance (*p*-value)**			
	**df**	**2 days**	**4 days**	**9 days**	**14 days**
Corrected model	3	0.000	0.000	0.000	0.000
Intercept	1	0.000	0.000	0.000	0.000
Temperature	1	0.000	0.022	0.000	0.002
Nutrients	1	0.000	0.000	0.000	0.000
Temperature ^∗^ nutrients	1	0.001	0.001	0.003	0.210
Error	62				
Total	66				
Corrected total	65				

*R Squared = 0.63 (Adjusted R Squared = 0.61) 2 days*

*R Squared = 0.62 (Adjusted R Squared = 0.60) 4 days*

*R Squared = 0.53 (Adjusted R Squared = 0.51) 9 days*

*R Squared = 0.41 (Adjusted R Squared = 0.38) 14 days*

*Results of the tests of significance (ANOVA) for the impact of incubation temperature and high-level nutrient addition on the % similarity of BCC between samples in Experiment 2 at Lake I-8 inlet. The *p*-value for the main effects (temperature and nutrients) and the interaction term is given for each day of the experiment. The overall model R-squared is given for each day of the experiment.*

Replicate high-level nutrient experiments tested the influence of initial BCC and initial water chemistry on BCC response to nutrient and temperature treatments. BCC was assessed after 14 days during the four experiments (Experiments 3a–d) conducted with bacteria and water from Lake I-8 inlet and Lake I-8 outlet in factorial combination. In these experiments, the initial bacterial community was the only statistically significant predictor of community similarity at the end of the experiment (**Table 3**), but nutrient addition was nearly significant (*p* = 0.058) and the interaction term between initial bacterial community and nutrients was significant (*p* = 0.007). For the inlet community (Experiments 4a,b), a two-way ANOVA indicated that temperature and nutrients were both statistically significant predictors of the similarity of community composition between treatments, with no significant interaction terms (**Table 4**). A comparable analysis of the outlet BCC similarities indicated that only nutrient addition was a significant predictor of BCC similarity between samples.

**Table 3:** **Experiments 3a–d**.

Dependent variable: community similarity		

	df	Significance (*p*-value)
Corrected model	14	0.008
Intercept	1	0.000
Bacteria source	1	0.000
Incubation water source	1	0.813
Temperature	1	0.182
Nutrients	1	0.058
Starting community ^∗^ nutrients	1	0.007
Error	105	
Total	120	
Corrected total	119	

*R Squared = 0.24 (Adjusted R Squared = 0.14)*

*Results of the tests of significance (ANOVA) for similarity of bacterial community composition on day 14 of four replicate high-level nutrient experiments in which the source of bacteria and incubation water was varied. The *p*-value for the main effects (bacteria source, water source, temperature, and nutrients) and the interaction term is given, along with the overall model R-squared.*

**Table 4:** **Experiments 4a,b**.

Dependent variable: community similarity					

		**Significance (*p*-value)**			
	**df**	inlet, 6 days	inlet, 11 days	outlet, 6 days	outlet, 11 days
Corrected model	3	0.000	0.000	0.000	0.049
Intercept	1	0.000	0.000	0.000	0.000
Temperature	1	0.000	0.001	0.217^∗^	0.088
Nutrients	1	0.000	0.000	0.000^∗^	0.037
Temperature ^∗^ nutrients	1	0.070	0.163	0.622	0.097

		**Degrees of freedom**			
Error df		51	42	62	41
Total df		55	46	66	45
Corrected total df		54	45	65	44

*R Squared = 0.44 (Adjusted R Squared = 0.41) inlet 6 days*

*R Squared = 0.66 (Adjusted R Squared = 0.64) outlet 6 days*

*R Squared = 0.49 (Adjusted R Squared = 0.46) inlet 11 days*

*R Squared = 0.18 (Adjusted R Squared = 0.12) outlet 11 days*

*Results of the tests of significance (ANOVA) for similarity of bacterial community composition after 6 and 11 days in two replicate high-level nutrient experiments conducted with bacteria and water from Lake I-8 inlet and Lake I-8 outlet. A Kruskal–Wallace test for outlet day 6 data (^∗^ in Table) confirmed non-significance with *p* = 0.619 for temperature and significance of *p* = 0.000 for nutrients. The *p*-value for the main effects (temperature and nutrients) and the interaction term is given for day 6 and day 11, along with the overall model R-squared for these days.*

## Discussion

Storm events can cause dramatic changes in arctic stream temperature and flushing of soil nutrients and DOC into streams (e.g., Kling et al., 2014), and these changes directly impact the growth and composition of bacterial communities (e.g., Judd et al., 2006). In this study we demonstrate that temperature affects the speed of response to nutrient subsidies, and show how bacterial communities respond to the individual and combined effect of these two drivers. Observations of BP at Toolik inlet showed that stream discharge, DOC, nutrient concentrations, and BP all changed in response to summer storm events (**Figure 2**). Water temperature was inversely related to stream discharge but did not appear to strongly constrain bacterial response to substrate additions of DOC and nutrients washed in from soil water at these temperatures, as has been previously observed (Adams et al., 2010). DOC also increased with peaks in discharge, but after an initial threefold increase with the first event, concentrations later in the summer varied by only ∼20% (**Figure 2**). Although the increases in DOC with storm events were lower later in the summer, bacteria could be sensitive to small variations in labile DOC supply particularly if storms also brought in pulses of ammonium from soil waters (**Figure 2**, day ∼220) that enable the bacteria to access previously unavailable carbon (Harder and Dijkhuizen, 1983; Kirchman, 1994; Gasol et al., 2009). However, the general covariance of discharge, nutrients, and DOC along with an inverse relationship with water temperature during the storm events makes it difficult to identify the main control of bacterial activity in natural systems, requiring the isolation of these factors in experiments.

### Mesocosms – Bacterial Activity

Bacterial activity responded rapidly to added nutrients in all experiments indicating strong nutrient limitation of bacterial communities in the Toolik Lake region. High-level nutrient treatments approximating maximum natural levels had a larger impact on BP than did elevated temperature. Low-level nutrient treatments also boosted BP, but the effect was limited compared to the effect of elevated temperature, which increased BP more quickly than did nutrients. While the high-level nutrient effect was observed across different bacterial communities collected at different times, it is possible that some of the response seen with the lower level of nutrients could be attributed to community-specific responses (as discussed below). This suggests that nutrient concentrations at the upper range of those found in the environment are required to overcome the restriction of low temperature on bacterial activity. For example, BP in high-level nutrient treatments reached similar magnitudes of activity regardless of temperature. This is in contrast with Vrede’s (2005) finding that low temperatures superseded any other control, including P limitation. Temperature did control the cellular response to added nutrients in all of our experiments, with higher temperatures increasing the speed at which bacterial activity increased. This is likely due to the increased speed of biochemical reactions and higher affinity for substrates at warmer temperatures (Nedwell, 1999), and increased response to nutrients at higher temperatures has been observed in other studies (Mindl et al., 2007; Säwström et al., 2007). The faster response of BP to nutrients at higher temperature occurred regardless of sampling location, initial community composition, or DOM concentration and source, indicating that this temperature–nutrient interaction may be a robust feature controlling bacterial activity in many aquatic environments.

Several studies have identified interacting effects of temperature and substrate on heterotrophic bacterial growth (reviewed in Pomeroy and Wiebe, 2001), and in many of these studies the effect of substrate addition on growth rate was enhanced at low-temperature and minimal at high temperatures, presumably because of reduced substrate affinity at low temperatures (e.g., Wiebe et al., 1992). This pattern was detectable in our low-level nutrient experiment in which increases in BP and cell-specific carbon uptake due to nutrient addition were greater at 12∘C than at 17∘C. However, these patterns were not detectable in the high-level nutrient experiments (**Figures 4** and 5), suggesting that storm-related nutrient pulses in arctic freshwaters must be of sufficient magnitude to overcome temperature limitation on bacterial growth and substrate affinity.

Cell numbers mirrored the corresponding BP measurements for all the experiments. For example, in the experiment with low-level nutrient addition, the cell-specific carbon uptake was more rapid at warmer temperatures, as found in other studies (White et al., 1991; Adams et al., 2010). In contrast, when greater amounts of nutrients were added, cell-specific carbon uptake was greater in fertilized treatments regardless of temperature. In the high-level nutrient experiments, cell-specific uptake was particularly high after 2 days and declined afterward once cell numbers reached a maximum. The same was not observed for the low-level nutrient experiment, possibly because the experiment was not extended until cell numbers reached a maximum or perhaps because of differences in initial community composition. Nevertheless, during the early phases of the experiments when cell numbers were still increasing, cell-specific carbon uptake reached a peak in all treatments and that peak was higher in incubations amended with nutrients.

Elevated cell-specific carbon uptake in nutrient treatments suggests elongation or growth in size of cells. Bacteria differ in size and shape by community and by growth stage (Lebaron et al., 2002). When nutrients are present, bacteria can delay cell division to take advantage of the resources and increase in size (Shiomi and Margolin, 2007). This interpretation is supported by observations in our experiments indicating a large number of long, filamentous bacteria appearing in the nutrient addition treatments. Apparently when large amounts of inorganic nutrients were added, both growth and reproduction of the filamentous portion of the bacterial community were stimulated, regardless of incubation temperature.

### Mesocosms – Communities

The fast response of bacteria to nutrient inputs was also observed in community dynamics. Both incubation temperature and nutrient addition changed community structure in as little as 2 days (**Tables 1** and **2**; **Figure 6**). The composition of communities created by nutrient addition steadily diverged from the controls in all experiments, especially in the 12∘C treatments (Supplementary Figure S1). However, in the 17∘C treatment BCC changed rapidly when nutrients were added, but then stayed similar over time. We interpret this to be a function of the higher rates of bacterial activity at warmer temperatures, which could lead to faster shifts in population or species dominance by the superior competitors under nutrient-enhanced conditions. This interpretation is supported by the elevated BP rate observed within 1–2 days in all experiments at 17∘C compared to 12∘C (**Figures 3** and **Figure 4**).

Despite the observed role of temperature in controlling the rate of change in BCC, under some conditions nutrients may be stronger drivers of community structure than temperature. This was demonstrated in the experiments using water and bacteria from I-8 inlet. This stream is not buffered by upstream lakes, and it had variable temperature and slightly higher nutrient concentrations than did I-8 outlet, which had more stable and higher temperatures than did I-8 inlet (Supplementary Tables S1 and S2). Communities from I-8 inlet responded to both temperature and nutrient additions (**Tables 2** and **4**), while communities from I-8 outlet responded to nutrients but not to temperature (**Table 4**). Bertoni et al. (2008) also found that shifts in BCC in response to nutrient additions were dependent on initial community composition, and this dependence may have reflected the *in situ* temperatures of different seasons. The different response of communities at the I-8 inlet and outlet to nutrient addition supports the hypothesis of community-specific nutrient limitation; in other words, communities that develop in separate, different habitats can respond uniquely to nutrient enrichment over time. These varied responses also suggest that natural site variability of temperature and nutrient concentrations are poor predictors of the stability of community composition in the face of rapid environmental change. Thus we suggest that community-specific responses to temperature and nutrients are not limited to BP and cell counts, but they also influence the stability of the community composition itself through competition between populations within the community.

Water temperature also impacts bacterial response to nutrients, particularly when these two factors are decoupled during storm events. As observed in the mesocosm experiments, colder temperatures can delay or diminish the response of bacterial activity to pulsed inorganic nutrients, and changing water temperature can shift bacterial communities to different populations than those stimulated by inorganic nutrients. Hall et al. (2009) also suggest that the bacterial response to temperature and nutrients changes seasonally, with summer and winter communities having different nutrient efficiencies relative to water temperature. According to this model, warm-adapted summer communities use nutrients more efficiently than winter communities. Here we show that the overall bacterial response to the interaction between temperature and nutrients is constrained by low temperatures, but that temperature constraints can be overcome by high levels of nutrients typical of storm-water pulses. We also show that population shifts resulting from differential responses to temperature or nutrients play an important role in the rapid shifts in community composition we observed.

## Conclusion

We demonstrate that aquatic bacteria in an arctic tundra environment can be nutrient limited, as predicted by theory, given that the processing of allochthonous, terrestrial carbon entering oligotrophic lakes, and streams requires additional nutrients for enzyme formation beyond regular cellular function. Experiments showed that inorganic nutrient additions and raised temperature increased bacterial productivity and growth rates rapidly, but nutrients above certain levels moderated the restrictive influence of low temperature on bacterial growth. Similarly, temperatures above certain levels drove very rapid shifts in BCC through the mechanism of enhanced activity accelerating the outcome of competition between species under new environmental conditions, such as altered nutrient concentrations during storm events. In addition, it appears that DOM characteristics (terrestrial versus algal, DOC concentrations) and initial environmental temperature and nutrient concentrations were poor predictors of the bacterial response to increased temperature and nutrients. We suggest that the resulting complex response to shifting temperature and nutrients occurs because different members of these communities are limited by different environmental factors. Consistent with this suggestion is our observation that shifts in community composition can occur very rapidly (∼2 days), and the resulting community can vary depending on the temperature and nutrient conditions during the pulse. A fast response of BP was also observed in the field following storm events during which discharge, ammonium, and BP peaked together. Bacterial communities in these habitats can respond rapidly to nutrient pulses through increased growth and shifting community composition, particularly at higher temperatures. Overall, these results suggest that under steady or slightly changing environmental conditions (e.g., temperature and nutrients), the initial BCC has a strong effect on the function of the community as measured by BP. As temperature or nutrient concentrations increase, BCC shifts rapidly but the influence of the initial community composition as a driver of community function diminishes, and the environmental controls on bacterial activity dominate. Thus the interaction between community composition (diversity) and function can shift rapidly as the environment changes, as exemplified by the often dramatic storm events that commonly affect aquatic ecosystems.

## Conflict of Interest Statement

The authors declare that the research was conducted in the absence of any commercial or financial relationships that could be construed as a potential conflict of interest.
