# Supplementary material for: Isolating the effects of storm events on arctic aquatic bacteria: temperature, nutrients, and community composition as controls on bacterial productivity
Source: Front Microbiol. 2015 Mar 31;6:250. doi: 10.3389/fmicb.2015.00250 (PMC4379936; doi:10.3389/fmicb.2015.00250)
Supplement: Supplementary file 1 [file presentation_1.PDF]

Isolating the effects of storm events on arctic aquatic bacteria: temperature, nutrients, and community composition as controls on bacterial productivity

Adams, Heather E., University of Michigan, Department of Ecology and Evolutionary Biology, Ann Arbor, USA, hea@umich.edu
*Crump, Byron C., Oregon State University, College of Earth, Ocean and Atmospheric Science, Corvallis, OR, USA, bcrump@coas.oregonstate.edu
Kling, George W., University of Michigan, Department of Ecology and Evolutionary Biology, Ann Arbor, USA, gwk@umich.edu

*Corresponding author
Byron C. Crump, College of Earth, Ocean and Atmospheric Science, Oregon State University, 104 CEOAS Admin Bldg., Corvallis, OR 97331, USA, bcrump@coas.oregonstate.edu

Running title: Aquatic bacterial nutrient and temperature limitation

Supplemental Table and Figures

Supplemental Table 1. Inorganic nutrient concentrations, temperature and chlorophyll *a* and (mean, minimum, maximum) in summers 2003-2007. Inorganic nutrient were sampled weekly from 15 June to 20 August in each year. Chl *a* was sampled 3-21 times a season. Temperature at I-8 inlet (2005-2006) and I-8 outlet (2004-2006) was measured continuously during summer with Onset HOBO temperature loggers. Temperature at Toolik inlet (2003-2007) was measured continuously during summer with a Campbell Scientific Model 247 conductivity and temperature probe connected to a Campbell Scientific CR510 datalogger. Temperature at Toolik outlet was measured weekly during summer with a digital thermometer (Fisherbrand Traceable).

site	year	NH4 (μM)			NO3 (μM)			PO4 (μM)			Temp. (°C)			Chl *a* (μg/L)		
		min	mean	max	min	mean	max	min	mean	max	min	mean	max	min	mean	max
I-8 inlet	2003	0.00	0.41	1.98	0.31	0.88	2.14	0.01	0.07	0.24	3.3	9.2	14.5	0.06	0.11	0.17
	2004	0.01	0.20	0.54	0.27	2.37	5.94	0.02	0.03	0.05	8.3	11.5	14.3	0.08	0.29	0.77
	2005	0.10	0.26	0.65	0.79	4.33	8.55	0.00	0.04	0.07	3.3	9.3	26.0	0.16	0.30	1.10
	2006	0.00	0.23	0.53	0.80	1.75	2.94	0.03	0.05	0.11	1.9	8.6	13.3	0.14	0.57	1.8
	2007	0.00	0.60	3.89	0.95	8.77	19.0	0.02	0.07	0.26	10.3	12.98	17.6	0.04	0.25	0.60
I-8 outlet	2003	0.00	0.24	1.52	0.03	0.26	0.73	0.02	0.07	0.33	5.8	10.5	15.2	0.22	0.79	1.64
	2004	0.12	0.27	0.67	0.12	0.33	0.97	0.02	0.03	0.04	7.1	13.4	20.4	0.29	1.09	2.22
	2005	0.12	0.31	0.49	0.00	0.30	0.70	0.00	0.04	0.06	8.3	12.4	26.6	0.41	1.08	2.28
	2006	0.00	0.16	1.06	0.03	0.25	0.68	0.02	0.04	0.10	4.7	11.0	14.0	0.54	1.53	2.49
	2007	0.00	0.30	0.73	0.07	0.17	0.46	0.03	0.05	0.10	10.5	15.7	18.4	0.34	1.15	2.24
Toolik inlet	2003	0.00	0.16	0.54	0.41	1.01	3.07	0.01	0.04	0.13	4.6	9.6	19.3	0.19	0.27	0.42
	2004	0.04	0.48	2.42	0.16	1.39	5.33	0.02	0.04	0.16	6.8	12.2	18.7	0.26	0.73	1.66
	2005	0.10	0.16	0.26	0.63	4.44	8.51	0.00	0.08	0.59	5.0	11.2	18.9	0.26	0.75	1.26
	2006	0.00	0.73	3.46	0.15	0.62	1.58	0.02	0.04	0.13	4.6	10.7	14.7	0.25	0.99	3.18
	2007	0.00	0.40	3.04	0.42	3.31	8.62	0.00	0.05	0.20	8.3	12.9	18.8	0.19	0.30	0.56
Toolik outlet	2004	0.05	0.21	0.66	0.00	0.04	0.16	0.02	0.04	0.10	11.7	13.9	15.9	0.12	0.94	1.65

Supplemental Table 2. Site conditions for experimental collections.

Location			Water Chemistry							
Site	Date	Time	Temp. (°C)	Chl *a* (µg/L)	DOC (µM)	NH_4 (µM)	PO_4 (µM)	NO_3 (µM)	TDN (µM)	TDP (µM)
I8 inlet	12 Jul 2005	10:15	4.7	0.09	707.1	0.65	0.04	0.79	17.7	N/A
I8 outlet	12 Jul 2005	10:50	9.2	0.77	515.4	0.37	0.02	0.70	13.2	N/A
I8 inlet	27 Jun 2006	11:00	7.8	0.36	778.7	0.53	0.03	0.83	18.8	0.12
I8 outlet	27 Jun 2006	10:15	11.6	2.49	697.3	N/A	0.03	0.15	15.6	0.11
I8 inlet	18 Jul 2006	9:38	10.1	0.41	463.8	0.47	0.04	2.54	14.1	0.15
I8 outlet	18 Jul 2006	10:32	12.4	2.10	575.4	0.26	0.03	0.06	12.9	0.13
Toolik inlet	22 Jun 2007	6:23	10.5	0.19	324.2	0.18	0.03	5.13	13.3	0.08

Supplemental Table 3. Details of the eight experiments. Each experiment included four treatments varying temperature and nutrients (N and P). Temperatures (12 °C and 17 °C) reflected community temperature optima previously detected in these habitats (Adams et al. 2010), and were similar to the average and maximum stream temperatures during summer. Nutrient treatments added inorganic nutrients to the background nutrient concentrations in sample waters (see Supplemental Table 1). Low-nutrient treatments had inorganic nutrients added to achieve concentrations of 1.5 µM NH_4NO_3 and 0.25 µM KH_2PO_4. High-nutrient treatments had inorganic nutrients added to achieve concentrations of 6.4 µM NH_4NO_3 and 0.45 µM KH_2PO_4. Bacteria inocula were diluted to approximately 10% of their volume with 0.2 µm filtered water at the start of the experiments. Incubations were conducted in either 4L LDPE cubitainers (Thermo Scientific) or 1L HDPE bottles (Nalgene).

Expt.	Treatments	Date of collection	Water source	Inoculum source	Nutrients added	Vol. (L)	Container	Duration	BP	Cell counts	DNA	Results
1	Low nutrients, temperature	22 Jun 07	Toolik inlet	Toolik inlet	Low	3.2	4L cubitainers	2 days (12 °C), 4 days (17 °C)	X	X	X	Table 1 Fig. 3, 6
2	High nutrients, temperature	27 Jun 06	I-8 inlet	I-8 inlet	High	3.0	4L cubitainers	14 days	X	X	X	Table 2, Fig. 4, 7
3a	High nutrients, temperature, source water, source community	12 Jul 05	I-8 inlet	I-8 inlet	High	1.25	4L cubitainers	14 days	X		X	Table 3, Fig. 5
3b		12 Jul 05	I-8 outlet	I-8 outlet	High	1.25	4L cubitainers	14 days	X		X	Table 3, Fig. 5
3c		12 Jul 05	I-8 outlet	I-8 inlet	High	1.25	4L cubitainers	14 days	X		X	Table 3, Fig. 5
3d		12 Jul 05	I-8 inlet	I-8 outlet	High	1.25	4L cubitainers	14 days	X		X	Table 3, Fig. 5
4a	High nutrients, temperature, source community	18 Jul 06	I-8 inlet	I-8 inlet	High	1.0	1 L bottles	11 days			X	Table 4
4b		18 Jul 06	I-8 outlet	I-8 outlet	High	1.0	1 L bottles	11 days			X	Table 4

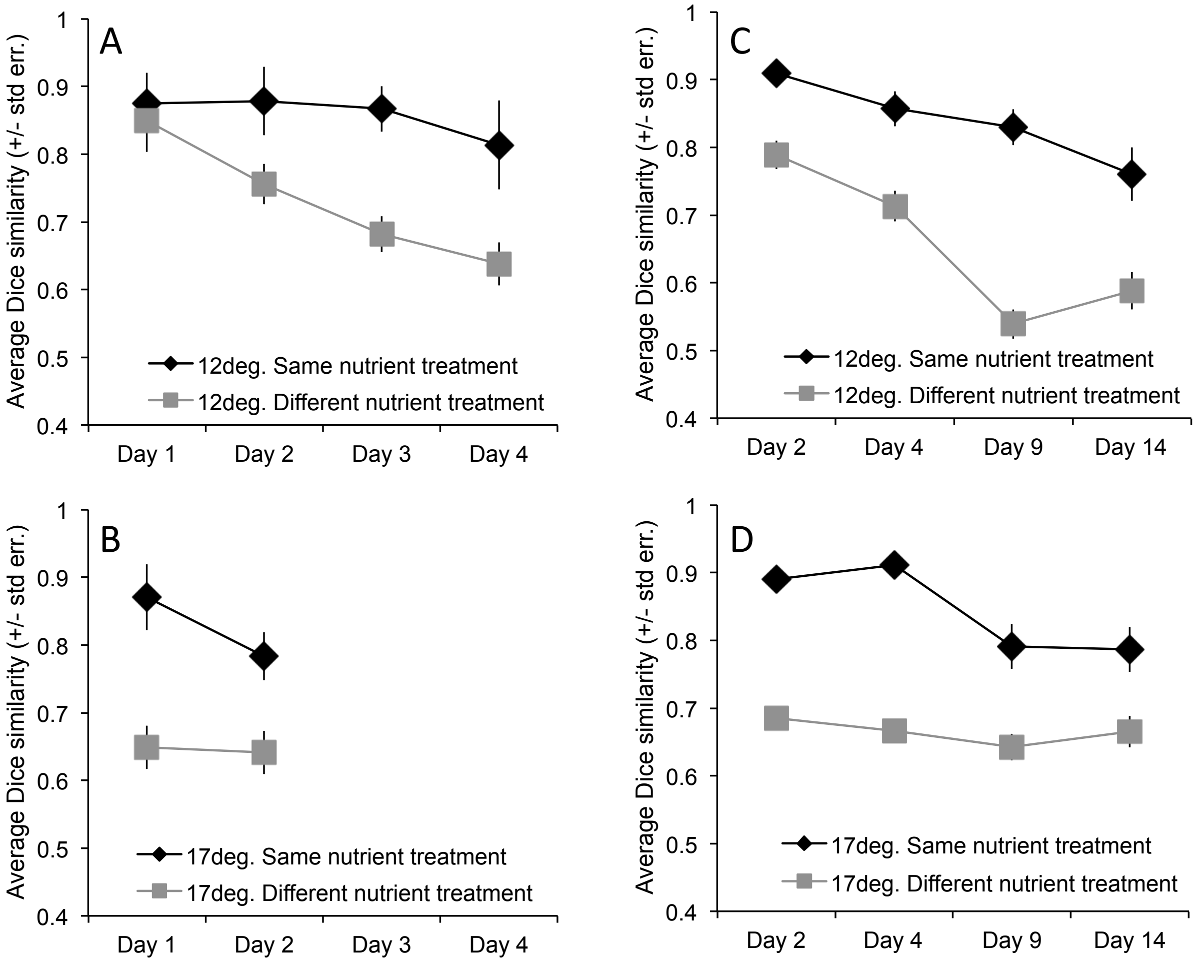

Supplemental Figure 1. Average Dice similarity of DGGE banding patterns within (♦) and between (■) different nutrient treatments for (A) the low-level nutrient experiment at 17 °C, (B) the low-level nutrient experiment at 12 °C, (C) the high-level nutrient experiment at 12 °C, and (D) the high-level nutrient experiment at 17 °C.
